# Establishment and effects of allograft and synthetic bone graft substitute treatment of a critical size metaphyseal bone defect model in the sheep femur

**DOI:** 10.1111/apm.12918

**Published:** 2019-01-30

**Authors:** Werner Hettwer, Peter F. Horstmann, Sabine Bischoff, Daniel Güllmar, Jürgen R. Reichenbach, Patrina S. P. Poh, Martijn van Griensven, Florian Gras, Michael Diefenbeck

**Affiliations:** ^1^ Musculoskeletal Tumor Section Department of Orthopedic Surgery Rigshospitalet University of Copenhagen Copenhagen Denmark; ^2^ Central Experimental Animal Facility University Hospital Jena Jena Germany; ^3^ Medical Physics Group Institute of Diagnostic and Interventional Radiology University Hospital Jena Jena Germany; ^4^ Experimental Trauma Surgery Department of Trauma Surgery Klinikum rechts der Isar Technical University of Munich Munich Germany; ^5^ Department of Trauma, Hand and Reconstructive Surgery University Hospital Jena Jena Germany; ^6^ BONESUPPORT AB Lund Sweden; ^7^ Scientific Consulting in Orthopaedic Surgery and Traumatology Hamburg Germany

**Keywords:** Animal model, bone graft substitute, calcium sulphate, critical bone defect, hydroxyapatite

## Abstract

Assessment of bone graft material efficacy is difficult in humans, since invasive methods like staged CT scans or biopsies are ethically unjustifiable. Therefore, we developed a novel large animal model for the verification of a potential transformation of synthetic bone graft substitutes into vital bone. The model combines multiple imaging methods with corresponding histology in standardized critical sized cancellous bone defect. Cylindrical bone voids (10 ml) were created in the medial femoral condyles of both hind legs (first surgery at right hind leg, second surgery 3 months later at left hind leg) in three merino‐wool sheep and either (i) left empty, filled with (ii) cancellous allograft bone or (iii) a synthetic, gentamicin eluting bone graft substitute. All samples were analysed with radiographs, MRI, μCT, DEXA and histology after sacrifice at 6 months. Unfilled defects only showed ingrowth of fibrous tissue, whereas good integration of the cancellous graft was seen in the allograft group. The bone graft substitute showed centripetal biodegradation and new trabecular bone formation in the periphery of the void as early as 3 months. μCT gave excellent insight into the structural changes within the defects, particularly progressive allograft incorporation and the bone graft substitute biodegradation process. MRI completed the picture by clearly visualizing soft tissue ingrowth into unfilled bone voids and presence of fluid collections. Histology was essential for verification of trabecular bone and osteoid formation. Conventional radiographs and DEXA could not differentiate details of the ongoing transformation process. This model appears well suited for detailed *in vivo* and *ex vivo* evaluation of bone graft substitute behaviour within large bone defects.

Bone grafts are thought to provide structural and/or cellular support to enhance biological repair and are considered essential for the restoration of larger bone defects arising from trauma or bone resections associated with tumour or infection surgery, and revision arthroplasty 1, 2, 3, 4. Typical biomaterials used to fill bone voids or defects in current clinical practice include autografts, allografts, demineralized bone matrix (DBM) and synthetic bone graft substitutes (BGS) 5. However, the efficacy of BGS is difficult to assess in humans; especially, transformation of a BGS into vital bone is challenging to prove. Highly sensitive imaging such as high resolution computed tomography (CT) with thin slices in accurate correlation with corresponding bone biopsies for histological analysis would be necessary. Both examinations, however, are unjustifiable in patients for basic research purposes due to the high radiation exposure and invasiveness of sample collection. Alternative imaging modalities with no or less radiation exposure, such as MRI or DEXA, can be difficult to interpret. Appropriate and well‐characterized animal models combining multiple adequate imaging methods with correspondingly aligned histology sections thus represent essential tools for basic research 6, 7. The aim of this pilot study was to develop a large animal model that would replicate a common clinical bone defect scenario as closely as possible and would also allow detailed analysis of the bone healing response to bone defect filling with a BGS in comparison to cancellous allograft or no filling at all. Since the resorption patterns of ceramic BGS are challenging to interpret and its transformation into bone are difficult to substantiate in clinical practice 8, we anticipated that the analysis of multiple different imaging modalities in correlation and accordance with histological findings would help to replicate, clarify and better understand clinical observations in humans.

## Materials and Methods

### Animals

Three healthy female merino‐wool sheep (age 2.5 years at first surgery) were used for this study and institutional guidelines regarding care of animals were followed. Animals were kept in groups and were fed standard animal chow. Approval of the animal protection committee of Thuringia, Germany (Reg. No. 15‐004/15) was obtained.

### Study design

Standardized bone voids were created in the medial femoral condyle of each hind leg using a staged approach with an interval of 3 months between both sides to reduce the number of test animals. The bone voids were treated with either: (i) no filling, (ii) filling with allograft or (iii) filling with a ceramic, gentamicin eluting BGS (CERAMENT^®^|G, BONESUPPORT AB, Lund, Sweden).

This bio‐composite consists of 60% calcium sulphate and 40% hydroxyapatite combined with the antibiotic gentamicin 17.5 mg/1 ml paste, initial porosity 20–40%, initial pore size 1 μm, increasing with calcium sulphate dissolution 9. The animals were sacrificed after 6 months, providing one sample with a follow‐up time 3 and 6 months from each respective treatment group for analysis (Table 1).

**Table 1:** Details of surgery and BV/TV results of μCT evaluation

Sheep	Side	Graft (in ml)	Sacrifice (in months)	BV (in cm³)	TV (in cm³)	BV/TV (×100%)
1	Right	Empty 10	6	3.45	9.83	35.10
1	Left	Allograft 11	3	4.86	11.48	42.33
2	Right	CERAMENT^®^|G 10	6	5.55	9.19	60.39
2	Left	CERAMENT^®^|G 11	3	6.01	14.71	40.86
3	Right	Allograft 10	6	3.22	10.32	31.20
3	Left	Empty 10	3	0.06	11.73	0.51

### Surgical technique

After induction of anaesthesia with ketamine (2 mg/kg i.m., Ketavet, Zoetis Deutschland GmbH, Berlin, Germany), midazolam (0.1–0.3 mg/kg i.m., Midazolam‐hameln, hameln pharma plus GmbH, Hameln, Germany) and propofol (0.2 mg/kg/h i.v., Narcofol, CP Pharma, Burgdorf, Germany) sheep were intubated for inhalation anaesthetics with isoflurane (1.5–2.5 Vol%, Isofluran CP, CP Pharma, Burgdorf, Germany) and propofol (0.2 mg/kg/h i.v., Narcofol, CP Pharma, Burgdorf, Germany). A single shot of fentanyl injection (0.1 mg/kg i.v., Fentadon, Albrecht GmbH, Aulendorf, Germany) for perioperative analgesia and ringer‐lactate infusion (10 ml/kg/h, Fresenius Kabi Deutschland GmbH, Bad Homburg, Germany) for volume substitution was given. Antibiotic prophylaxis was administered using ampicillin (10 mg/kg i.v. every 12 h, Ampi Dry, Veyx, Schwarzenborn, Germany) and enrofloxacin (2.5 mg/kg, s.c./i.v. per day, Baytril, Bayer Vital, Leverkusen, Germany).

Surgical site disinfection (Braunoderm, B. Braun Melsungen AG, Melsungen, Germany) and draping with a sterile incision foil was performed, according to clinical standards. An 8 cm skin incision (starting 2 cm proximal and anterior to the medial femoral epicondyle) and splitting of fascia and adductor muscles were performed to access the joint capsule. After capsulotomy, a cancellous bone cylinder with a depth of 2 cm was removed from the medial femoral condyle, using a 25 mm custom‐made trephine, resulting in a perfectly cylindrical, contained bone defect with an approximate volume of 10 ml (*V* = π × *r*² × h = 3.1415 × (1.25 cm)^2^ × 2 cm = 9.81 cm³). The bone defects were treated according to the planned study group allocation (Table 1): (i) Unfilled voids were simply covered by the overlying soft tissues closed in multiple layers. (ii) Fresh frozen allograft, retained from defect creation in sheep 1 and 2, was thawed at the time of the surgery, morselized and impacted into the bone voids with a bone tamp. (iii) The BGS CERAMENT^®^|G (BONESUPPORT AB, Lund, Sweden) was prepared according to the manufacturer's instructions, injected and compressed into the respective bone voids. Joint capsule, fascia and subcutaneous tissues were closed with interrupted Vicryl 2‐0 (Ethicon, Norderstedt, Germany). The skin was closed with an intradermal horizontal mattress suture (3‐0 Vicryl‐rapid), followed by application of an occlusive skin adhesive consisting of 2‐octyl cyanoacrylate (Liquiband Flex, Advanced Medical, Winsford, UK). A sterile wound dressing and well‐padded scotch cast (3M, Neuss, Germany) were applied.

### Post‐operative care

Antibiotic prophylaxis was continued for 3 days. For post‐operative pain control, buprenorphine (0.02 mg/kg, i.v., every 12 h, Buprenovet, Bayer Vital, Leverkusen, Germany) and metamizole (25 mg/kg, i.v., every 12 h, Metamizol‐WDT, Garbsen, Germany) were used.

Cast immobilization was maintained for 14 days to protect the skin incision and to secure wound healing.

### Sacrifice

At 6 months, the animals were euthanized with an overdose of pentobarbital (60 mg/kg, i.v. Pentobarbital, Merial, Söldnermoos, Germany).

### Radiographs

Conventional radiographs in anterior–posterior (ap) and lateral (lat) projection were obtained immediately after surgery, at monthly intervals and *ex vivo* after sacrifice corresponding to the 3‐ and 6‐month follow‐up time points using a Philips X‐ray Integris H3000 digital radiography system.

### MRI (*ex vivo*)

The hind legs were ex‐articulated at the hip joints, transected at the distal tibia and vacuum sealed in a plastic bag using a vacuum device (CASO 1340 VC10, CASO‐Germany, Arnsberg, Germany) for immediate transfer to the MRI department. The sealed specimens were scanned within 1 hour after sacrifice, using a 3 T whole‐body MR scanner (Magnetom PRISMA fit, Siemens Healthineers, Erlangen, Germany) and a 15‐channel transmit/receive knee coil. The imaging protocol included T1‐ and T2‐weighted 2D turbo‐spin‐echo sequences in transverse and coronar orientation (Table 2). Additionally, a TIRM sequence for fat suppression was acquired in coronar orientation only. The acquisition time per protocol run (one knee) was approximately 25 min.

**Table 2:** Details of MRI settings

	TE in ms	TR in ms	Slices	Slices thickness in mm	FOV in mm	In‐plane resolution in mm²
T1w‐TSE	12	684	25	3	160	0.4 × 0.4
T2w‐TSE	103	6310	25	3	160	0.4 × 0.4
TIRM	30	3060 (TI 200)	25	3	160	0.5 × 0.5

### μCT

After the MRI scans, the hind legs were dissected for macroscopic analysis to obtain cubic bone samples of 27 cm^3^ (3 cm × 3 cm × 3 cm) in size, containing the entire defect and the surrounding host bone. All samples were scanned in 70% ethanol solution using a μ‐CT (Skyscan 1176, Kontich, Belgium) at 90 kV, 277 μA with a 0.1 mm Cu filter. Image projections were acquired at a resolution of 35 μm every 0.7° rotation step over 360° rotation around the vertical axis and processed with a volumetric reconstruction software (NRecon, version 2.0.4) to determine ratios of total bone/biomaterial volume (BV) to total defect volume (TV) using the built‐in bone morphology algorithm of the CT Analyzer software version 1.13 (Bruker, Kontich, Belgium).

### DEXA

The cubic bone samples were kept in 5% neutral buffered formalin for at least 10 days, dehydrated with an increasing ethanol series, impregnated with a 1:1 mixture of absolute ethanol and Technovit embedded into a 100% methacrylate‐based resin (Technovit 7200VLC, Heraeus Kulzer, Wehrheim/Ts, Germany) without prior decalcification. Samples were transferred to the DEXA‐facility for scanning on a Norland XR 46 (Norland Corp. Fort Atkinson, WI, USA) bone densitometer using the *small subject* scan option with a scan speed of 20 mm/s and pixel‐size of 0.5 × 0.5 mm. Scanning direction was along the longitudinal axis of the bone defects.

### Histology

After the Dexa scan, axial 200 μm sections were prepared using an EXAKT 300 diamond band saw, ground to a thickness of 80–100 μm with the EXAKT 400CS grinding system and stained with haematoxylin–eosin (HE) without removing the polymethacrylate. Bone morphology surrounding the defect was analysed using ZEISS ZEN Imaging Software (Carl‐Zeiss GmbH, Jena, Germany).

## Results

### Animals

All three animals tolerated both surgeries without any peri‐ or post‐operative complication and showed normal behaviour, physiological range of hind leg motion and full weight bearing 3 weeks after surgery.

### Radiographs

**Figure 1:**
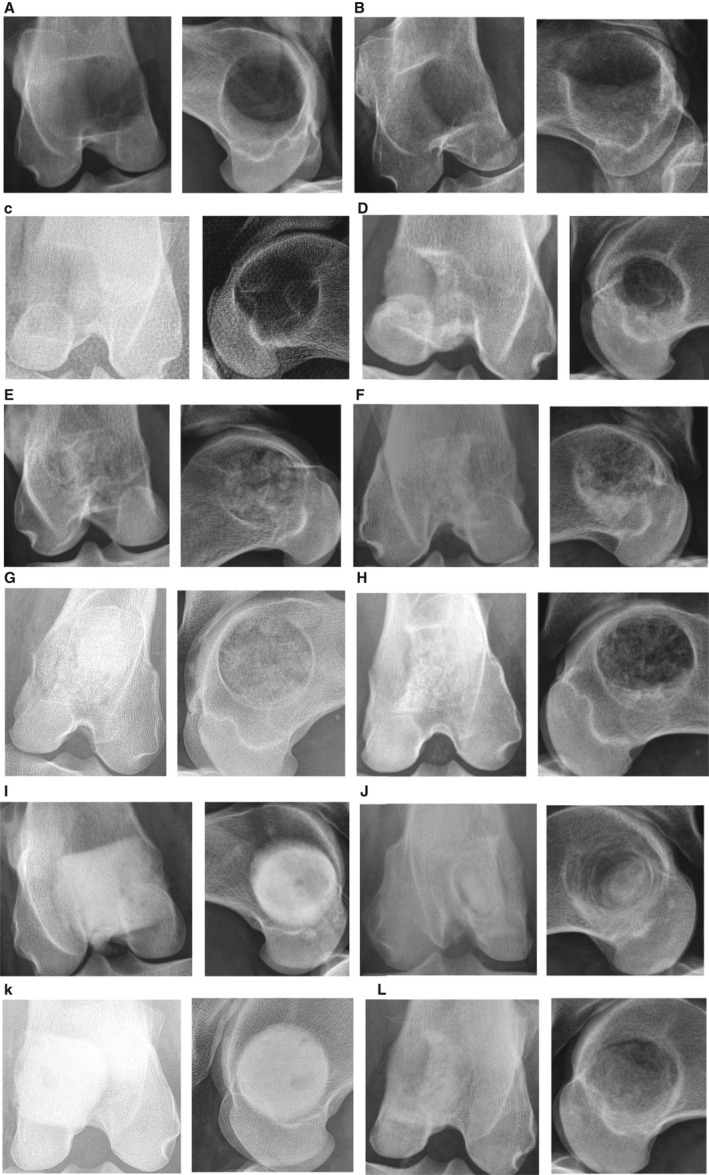
Radiographs of the distal femur in anterior–posterior and lateral view post‐operative and at the final follow‐up at 3 or 6 months. (A) Unfilled (empty): post‐operative. (B) Unfilled: follow‐up at 3 months. (C) Unfilled (empty): post‐operative. (D) Unfilled: follow‐up at 6 months. (e) Allograft: post‐operative. (F) Allograft: follow‐up at 3 months. (g) Allograft: post‐operative. (H) Allograft: follow‐up at 6 months. (I) BGS: post‐operative. (J) BGS: follow‐up at 3 months. (K) BGS: post‐operative. (L) BGS: follow‐up at 6 months.

The unfilled bone void appeared radiolucent and almost unchanged at 3 months after surgery (Fig. 1B). At 6 months, the circumference of the drill hole appeared sclerotic, whereas the void itself remained radiolucent (Fig. 1D). Cancellous bone allograft (CBA), recognizable inside the bone voids on post‐operative radiographs as a radio‐dense, trabecular structure (Fig. 1E and G), showed confluence of this trabecular structure, coinciding with a higher overall radio‐density at 3 months (Fig. 1F) and a less dense trabecular structure with some radiolucent areas in the 6 months sample (Fig. 1H). Bone voids treated with BGS displayed the typical immediate post‐operative appearance of homogeneous fill with radiopaque material (Fig. 1I and K). Follow‐up samples at 3 months showed a radio‐dense marble‐like central structure surrounded by a radiolucent area with onion‐like layers of radio‐dense material in its periphery (Fig 1J). After 6 months, the void appeared more uniform and less radio‐dense (Fig. 1L).

### Macroscopic evaluation

The samples of the different treatment groups showed distinct macroscopic characteristics. Both unfilled bone voids (i) were completely filled with fibrotic soft tissue. In the allograft samples (ii), cancellous bone was visible at the opening of the void. The voids filled with BGS (iii) showed a thin soft tissue layer covering the top of the void. Beneath this layer, the synthetic BGS was visible.

### μCT

**Figure 2:**
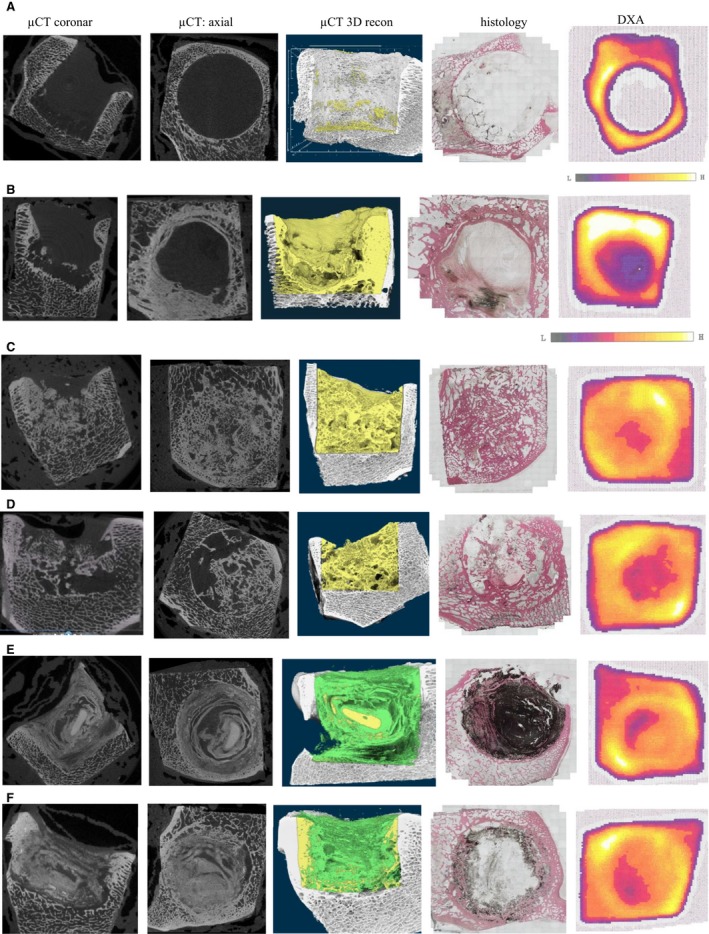
μCT images of the distal femur in coronar and axial view at the final follow‐up at 3 or 6 months. 3D reconstruction of μCT scans, colour‐coded according to grey scales, Histology in HE stain, DXA scans of the distal femur at the final follow‐up at 3 or 6 months. (A) Unfilled (empty): follow‐up at 3 months. (B) Unfilled (empty): follow‐up at 6 months. (C) allograft: follow‐up at 3 months. (D) Allograft: follow‐up at 6 months. (E) BGS: follow‐up at 3 months. (F) Bone Graft Substitute: follow‐up at 6 months.

Grey scale images and 3‐D reconstructed models demonstrated that the unfilled bone defect clearly remained devoid of any newly formed bone at 3 months (BV/TV ratio = 0.51%), (Fig. 2A, Table 1). At 6 months, dense, sclerotic bone with very narrow trabeculae was visible around the circumference of the drill hole and a calcified bony structure had formed predominantly at the bottom of the void (Fig. 2B). The allograft sample displayed complete filling with a dense, trabecular network of calcified material. Trabecular connections to the surrounding host bone were clearly visible at 3 months (Fig. 2C) with a BV/TV ratio of 42.33% (Table 1). In the 6 months sample a rarefication of the trabecula structure was noticed with a BV/TV ratio of 31.20% (Fig. 2D, Table 1). The 3‐month BGS‐sample showed a homogeneous, radio‐dense core in the centre of the void (Fig. 2E), surrounded by onion‐like layers of alternatingly radiolucent or radiopaque appearance, which had no similarity to trabecular bone. At 6 months, the material inside the void no longer revealed a defined structure (Fig. 2F); however, some trabecular bone appeared to have grown into the periphery of the void, as identified especially from the axial μCT view and the 3D reconstruction. While overall BV/TV ratios in the unfilled and allograft voids decreased from 3–6 months, an increased BV/TV ratio was noted in the BGS group (40.86–60.39%) (Table 1).

### Histology

HE‐stained sections of the unfilled bone voids showed no evidence of calcified material at 3 months (Fig. 2A). At 6 months, a rim of dense, sclerotic bone around the defect was apparent (Fig. 2B), protruding into the defect in an osteophyte‐like fashion along one‐quarter of the circumference. The CBA‐void displayed a well‐organized network of dense trabecular bone tissue with ample interconnections with the surrounding bone at 3 months (Fig. 2C). Nuclei were clearly identifiable as a sign of bone vitality. In the 6‐month sample, the trabecular network appeared less dense and less homogeneous (Fig. 2D). In some areas, no bony structure was seen; in other regions thick, hypertrophic trabeculae were visible. The BGS appeared black, with a fine‐grained structure in the HE stains. In the 3‐month sample, the biodegradation process of the BGS had started from the periphery towards the centre of the void (Fig. 2E). Osteoid could be clearly identified between the layers of the BGS, together with some new trabecular bone that had appeared at the periphery of the void. In the 6‐month sample, this ring of trabecular bone had increased in thickness, expanding towards the centre (Fig. 2F). In this section, almost no remnants of the BGS were visible, which is probably due to loss during the preparation process of this particular specimen.

### DEXA

In the unfilled bone void at 3 months, no bone mineral density (BMD) could be measured at the drill hole (Fig. 2A) as most of the trabecular bone adjacent to the bottom of the void had been removed in this particular specimen. The unfilled void at 6 months showed the typical appearance of low BMD (Fig. 2B). BMD of the 3‐month sample was comparable to the density of normal surrounding trabecular bone (Fig. 2C). Only the centre of the void had a lower BMD. At 6 months, BMD was comparatively lower (Fig. 2D). The 3‐month BGS‐void also showed BMD similar to the surrounding bone, with one region of lower density (Fig. 2E). The appearance of the 6‐month sample on the DEXA scan was almost comparable to the 3‐month sample.

### MRI

**Figure 3:**
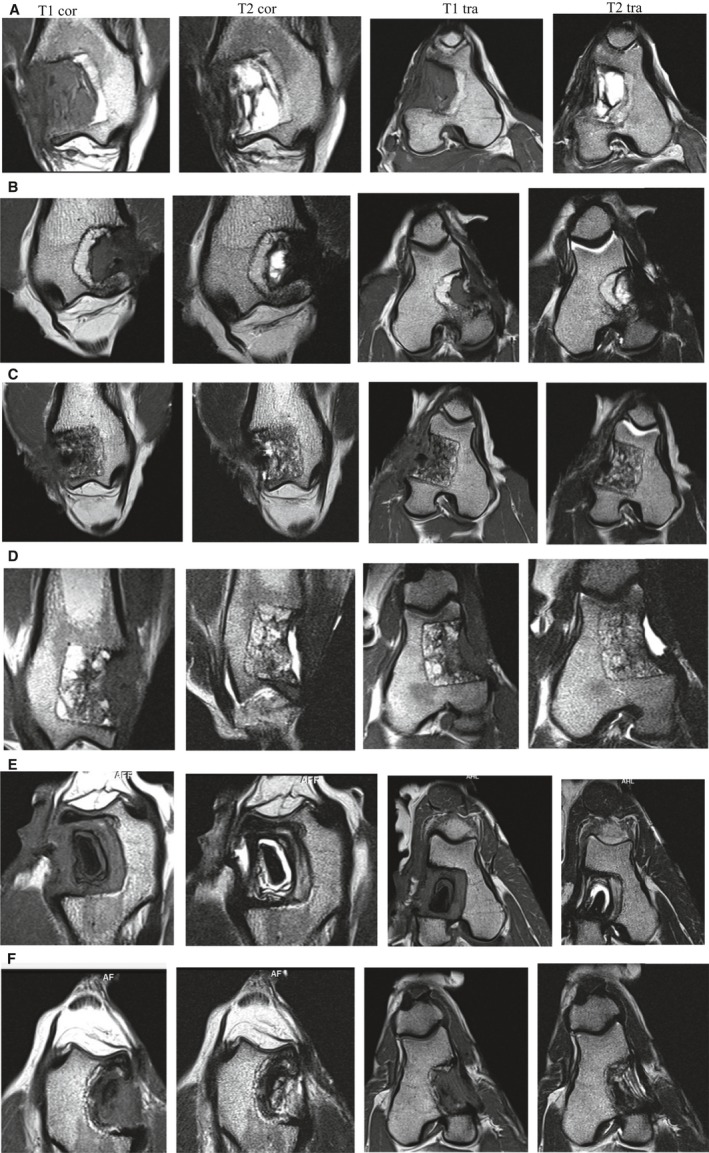
MRI images of the distal femur in coronar and transversal view (T1 and T2 weighting at the final follow‐up at 3 or 6 months. (A) unfilled (empty): follow‐up at 3 months. (B) unfilled (empty): follow‐up at 6 months. (C) Allograft: follow‐up at 3 months. (D) allograft: follow‐up at 6 months. (E) BGS: follow‐up at 3 months. (F) BGS: follow‐up at 6 months.

At 3 months, T1w images of the unfilled bone void clearly showed homogeneously appearing, slightly hypointense tissue throughout the entire drill hole (Fig. 3A). On the corresponding T2w images, this tissue appeared hyperintense, compatible with soft tissue and collections of liquid. In the 6‐month sample, a thin hypointense lamella was visible between the regenerated and the original bone on both MR sequences and more than two‐thirds of the void were filled with hypodense material, again compatible with soft tissue (Fig. 3B). The CBA was recognizable in the bone voids at 3 (Fig. 3C) and 6 months (Fig. 3D) by a hypointense irregular granular structure on both image sequences. Some small hyperintense areas on the T2w images most likely represent fluid between the cancellous graft. At 3 months, ingrowth of soft tissue towards the top of the void was apparent, especially on the T1w images (hypointense material in connection with the soft tissue around the void). In the 6 months sample, a hyperintense fluid depot was visible at the top part of the void (Fig. 3D). The BGS displayed a rather unique MRI morphology, probably reflecting the biodegradation process of the material. In the 3‐month sample, a uniform, hypointense core structure of spheroid shape was seen in the centre of the void on both T1w and T2w images, resembling a “marble” (Fig. 3E). This hypointense central structure was surrounded by layers arranged in an onion‐like fashion, with mixed hyper‐ and hypointense signal intensities on both, differently weighted MR images. The latter observation might correspond to layers of degraded BGS with fluid collections in between. In the 6‐month sample, the hypointense core had become much smaller compared to the 3‐month sample (Fig. 3F). The material, now occupying the void, appeared more or less entirely homogeneous on the T1w images and the appearance of the layers on the T2w images had become thinner with less fluid depots being visible.

## Discussion

### Model

To be comparable with clinical scenarios, appropriate animal models should mimic clinical procedures as closely as possible. As sheep are considered to exhibit similar healing rates of bone defects as humans 6, they have previously been chosen to establish useful models for fracture healing and bone remodelling activity 7. Also, due to their size, surgical techniques similar or even identical to human surgery can be employed. Many ovine animal models exist to evaluate various aspects of bone healing, ranging from bone chamber implants 10, confined defects in cancellous bone 11, 12 to segmental defects 13, 14. In order to replicate the clinical scenario of a metaphyseal bone resection, the largest possible standardized bone voids amenable to this bone region were created with a diameter of 25 mm and a depth of 20 mm. The resulting volume of approximately 9.8 ml corresponds very well to clinical studies, where the typical volume of BGS applied was 10 ml 15. Other epi/metaphyseal bone defect models have been described; however, the defect volumes in these models are typically a lot smaller and not entirely critical. Malhotra et al. evaluated drill holes in the medial distal femoral epiphysis and medial proximal tibial epiphysis of sheep with diameters of 8, 11 and 14 mm and 2.5 cm depth, resulting in defect volumes of 1.25 ml, 2.38 ml and 3.85 ml respectively 16. They concluded that increasing the bone defect diameter resulted in less complete healing in their model. However, they also found evidence of partial bone regeneration in each defect as early as 1 month after surgery. In the 3.85 ml defects, the BV/TV ratio was 22% at 4 weeks 16. In our present study, μCT and histology of the unfilled voids at 3 and 6 months clearly demonstrated no spontaneous healing within the bone voids, proving them to be truly “critical sized defects”. Therefore, our model appears suitable and valid for evaluating bone regeneration mediated by bone grafts and BGS. Fresh frozen allograft was chosen as bone void filler in the positive control group, since allograft is increasingly used in the clinical setting compared to autograft. With the demographic change towards an ageing population, the amount and quality of the harvested autograft is limited and alternative graft materials are needed.

### Surgical aspects

We specifically chose the distal femur to create a fully contained cancellous bone void without direct connection to the intramedullary (IM) canal as the IM canal is a potential source of bone marrow derived stem cells (BMSC), which could support bone healing 17 and might positively influence the results. On the other hand, graft material is more difficult to contain within defects extending into the medullary canal, which in turn could affect the results and would increase variability of defect and graft material volumes. A medial approach was chosen for an ease surgical access as well as to provide good soft tissue coverage over the respective bone defects. Further advantages of this defect model include its inherent stability without requiring additional fixation, which would interfere with the subsequent diagnostic work‐up.

### Imaging modalities

Conclusive evaluation of bone regeneration in unfilled bone voids or voids filled with CBA or BGS is difficult when solely based on radiographs. Radiographic grading scales are often used by independent and blinded observers for analyses of bone substitute integration in clinical settings 16, 18. However, this approach relies on qualitative and subjective impressions, which are prone to bias and low inter‐observer agreement. We therefore also acquired μCT scans, which permit a quantitative, observer‐independent evaluation, using μCT‐based biomaterial/bone volume to total volume (BV/TV) ratios (Table 1). Each treatment group exhibited clearly distinctive radiographic, μCT, MRI and histological follow‐up findings with characteristic and concordant imaging and microscopy features. Unfilled voids only showed ingrowth of fibrous tissue at 3 and 6 months but no evidence of spontaneous bone regeneration (despite complete containment by adjacent vital bone tissue), which indicates that these defects were truly critical. The voids filled with CBA (II) showed good integration of the cancellous graft at 3 months, confirming that CBA may be rightly considered an effective material for bone void filling 19. Rarefication of the trabecular structure, noticed on μCT images with a reduction of the BV/TV ratio at 6 months, could partly be due to the ongoing physiological process of bone remodelling and/or graft resorption. The results observed in the voids filled with the hydroxyapatite/CaS BGS clearly illustrate the value of a comparison and correlation of the different imaging modalities. Compared to conventional radiographs, μCT and MRI provide much clearer insight into the details of the ongoing centripetal BGS biodegradation process, with only histology being able to identify the presence of osteoid within the layers and to confirm new trabecular bone formation in the periphery of the void as early as 3 months. At 6 months further expansion of peripheral trabecular bone formation towards the centre of the void was clearly evident on μCT and histology. Overall, BMD measurements with DEXA correlated well with the above‐mentioned imaging modalities; however, they could not contribute to the detailed analysis of the transformation process.

### Clinical relevance

Our model provides objective evidence that reconstruction of a critical sized metaphyseal bone defect with CBA or a BGS leads to verifiable regeneration of new viable bone within such defects, whereas leaving said defects empty does not. It also shows that CBA‐ and BGS‐mediated bone regeneration appears to follow quite different pathways with distinctive and characteristic imaging and histology features. Since these features are highly consistent with the radiological biodegradation and bone formation patterns of CBA and BGS observed in a clinical series of patients with chronic osteomyelitis, treated with the same BGS 20, the comparison of the various imaging modalities in correlation with histology used in our model, favourably contributes to further understanding of the biological remodelling of cancellous bone allograft and the BGS.

The ability of our model to reproduce imaging features comparable to clinical practice and to assist with objective interpretation and quantitative estimation of the amount of vital bone being present in a bone defect, indicates that it may not only allow assessment and comparison of bone defect reconstruction methods under clinically relevant and realistic conditions but that it may even be suitable for clinical benchmarking of various bone defect reconstruction methods and bone reconstruction compositions. Finally, our findings also suggest that filling of critical sized bone voids with an appropriate material may indeed be necessary to avoid fibrotic tissue ingrowth and to promote generation of vital bone in order to contribute to lowering the risk of subsequent pathologic fractures though large critical sized bone defects in clinically relevant anatomic locations.

### Limitations

The limitations of our study include the small number of specimens included in this pilot study and the limited number of observation time points over a fairly short observation period, during which the remodelling process of the BGS was obviously not completed. Clearly, an analysis of a complete transformation process of the BGS would require substantially longer follow‐up periods of at least 12 months or probably even more. Equally, comprehensive sequential imaging and histological investigations performed at much shorter intervals would be desirable for an improved and more detailed documentation and understanding of the degradation and remodelling processes. A larger number of animals could certainly overcome these limitations, but would have to be balanced against costs and availability of the respective resources, which both remain relevant constraints. Finally, the model uses a biomechanical stable situation. In the clinical situation, large defects are often associated with biomechanical instability. Therefore, this model cannot be applied for instable bone defects, like non‐unions or segmental defects.

In conclusion**,** a standardized large animal model with a critical sized cancellous bone defect that allows objective assessment and comparison of various bone defect reconstruction methods under clinically relevant and realistic conditions was established. This model appears well suited for detailed *in vivo* and *ex vivo* evaluation of BGS behaviour within large bone defects with a comprehensive panoply of *in vivo* and *ex vivo* diagnostic methods. This pilot study represents an important initial step towards establishing a clinically relevant large animal model that allows thorough investigation of BGS‐mediated bone defect regeneration, in‐depth analysis of its interaction with host bone and accurate documentation of the resulting new bone formation over time.

We thank Prof. G. Schneider, Dr. A. Enkelmann and Mrs. S. Voigt from the Biomaterials Lab of the clinic for Otorhinolaryngology, University Hospital Jena for the preparation of the histological specimen.
